# Enzymatic- and temperature-sensitive controlled release of ultrasmall superparamagnetic iron oxides (USPIOs)

**DOI:** 10.1186/1477-3155-9-7

**Published:** 2011-02-27

**Authors:** Shann S Yu, Randy L Scherer, Ryan A Ortega, Charleson S Bell, Conlin P O'Neil, Jeffrey A Hubbell, Todd D Giorgio

**Affiliations:** 1 Department of Biomedical Engineering, Vanderbilt University; Nashville, Tennessee, USA; 2 Vanderbilt Institute for Nanoscale Science and Engineering, Vanderbilt University; Nashville, Tennessee, USA; 3 Interdisciplinary Program in Materials Science, Vanderbilt University; Nashville, Tennessee, USA; 4 Integrative Biosciences Institute, École Polytechnique Fédérale de Lausanne, Lausanne, Switzerland

## Abstract

**Background:**

Drug and contrast agent delivery systems that achieve controlled release in the presence of enzymatic activity are becoming increasingly important, as enzymatic activity is a hallmark of a wide array of diseases, including cancer and atherosclerosis. Here, we have synthesized clusters of ultrasmall superparamagnetic iron oxides (USPIOs) that sense enzymatic activity for applications in magnetic resonance imaging (MRI). To achieve this goal, we utilize amphiphilic poly(propylene sulfide)-*bl*-poly(ethylene glycol) (PPS-b-PEG) copolymers, which are known to have excellent properties for smart delivery of drug and siRNA.

**Results:**

Monodisperse PPS polymers were synthesized by anionic ring opening polymerization of propylene sulfide, and were sequentially reacted with commercially available heterobifunctional PEG reagents and then ssDNA sequences to fashion biofunctional PPS-bl-PEG copolymers. They were then combined with hydrophobic 12 nm USPIO cores in the thin-film hydration method to produce ssDNA-displaying USPIO micelles. Micelle populations displaying complementary ssDNA sequences were mixed to induce crosslinking of the USPIO micelles. By design, these crosslinking sequences contained an EcoRV cleavage site. Treatment of the clusters with EcoRV results in a loss of R_2 _negative contrast in the system. Further, the USPIO clusters demonstrate temperature sensitivity as evidenced by their reversible dispersion at ~75°C and re-clustering following return to room temperature.

**Conclusions:**

This work demonstrates proof of concept of an enzymatically-actuatable and thermoresponsive system for dynamic biosensing applications. The platform exhibits controlled release of nanoparticles leading to changes in magnetic relaxation, enabling detection of enzymatic activity. Further, the presented functionalization scheme extends the scope of potential applications for PPS-b-PEG. Combined with previous findings using this polymer platform that demonstrate controlled drug release in oxidative environments, smart theranostic applications combining drug delivery with imaging of platform localization are within reach. The modular design of these USPIO nanoclusters enables future development of platforms for imaging and drug delivery targeted towards proteolytic activity in tumors and in advanced atherosclerotic plaques.

## Background

Enzymatic activity is understood to be a hallmark of various diseases, including cancer and atherosclerosis [1,2]. Consequently, enzymatically-sensitive drug- and contrast agent-delivery platforms are of great interest in medical areas. Enzymatically-sensitive controlled release platforms have been previously investigated for drug delivery [3-5]. While they have also been investigated for molecular imaging, most of these efforts have been concentrated in the areas of optical imaging and nuclear imaging [1,6,7]. In many cases, these techniques are disadvantageous for *in vivo *applications because optical imaging is significantly limited by tissue autofluorescence and light absorbance, while nuclear imaging can expose the patient to relatively high doses of ionizing radiation. Magnetic resonance imaging (MRI) is not limited by these issues and provides the advantages of high spatial resolution and excellent soft tissue contrast. Only a few examples of enzymatically-sensitive platforms for MRI applications have been previously reported, as reviewed elsewhere [1].

Ultrasmall superparamagnetic iron oxides (USPIOs) have been widely investigated for applications as MRI contrast agents and for probing intermolecular interactions due to their strong T2 magnetic relaxation properties [8-11]. As contrast agents, USPIOs have unique characteristics, including high detection sensitivity, relatively low toxicity, and the potential for long circulation half-lives [12,13]. To produce USPIOs of uniform composition, size, and physical properties, thermal decomposition synthesis is preferred, but the process yields USPIO cores coated with a layer of the hydrophobic surfactant oleic acid [14].

Especially for our applications, biocompatible, bioactive USPIO-based contrast agents must exhibit solubility and stability in water and, in many cases, to display ligands such as whole proteins, peptides, or nucleic acids. In order to achieve this goal, a modular approach for functionalizing USPIOs is generally followed. Various methods for rendering USPIOs water-soluble are well-documented, including covalent methods such as silanization or the formation of micelles with polymers or phospholipids [8,15-18]. A wide range of techniques in bioconjugate chemistry can then be used to immobilize bioactive ligands onto the USPIO surface [19].

Some USPIO formulations are biocompatible and have been clinically approved for human use, such as Feridex and GastroMARK [20-22]. However, nanoparticle biocompatibility is largely determined by surface properties, independent of USPIO characteristics. Because of this, *in vivo *biodistribution must be determined for each unique formulation [23-26].

In recent years, the encapsulation of USPIOs in micellar structures by self-assembly with amphiphilic PEG-containing block copolymers has received attention [17,27,28]. Recently, extensive studies by the Hubbell group have shown that amphiphilic block copolymers of PEG and the hydrophobic poly(propylene sulfide) (PPS) can be used to generate micellar and multilamellar structures for drug delivery applications [29,30]. These copolymers have received interest for their unique characteristics, including a PPS block capable of undergoing a hydrophobic-to-hydrophilic transition in oxidative environments, resulting in environmentally-sensitive drug release [30,31]. Though previously uninvestigated as a USPIO coating, the PEG-PPS copolymers display material properties that presumably enable the construction of novel oxidation-responsive "theranostic" (therapeutic-diagnostic) agents in the near future. To add to these properties, PEG-PPS copolymers have been successfully tagged with bioactive ligands such as peptides for actively targeted drug delivery [32]. Here, we report the broader utility of the PEG-PPS copolymer platform through the synthesis of PPS-PEG-ssDNA constructs, and the self-assembly of these constructs onto highly monodisperse USPIO cores to generate multifunctional magnetofluorescent nanoparticles.

To demonstrate the applicability of the approach, these novel ssDNA-tagged USPIOs will then be assessed as magnetic relaxation switches (MRS) [33]. The MRS concept indicates that clustering of USPIOs leads to a significant increase in R_2 _relaxivity of the USPIOs, while redispersion of the USPIOs returns R_2 _to baseline levels. The MRS label originated from the behavior of the system as a nanosensor capable of being turned on or off in the presence of a specific environmental stimulus, which, in this study, is restriction enzyme activity. Complementary populations of ssDNA-USPIOs were mixed to form self-assembled clusters. These clusters were subjected to restriction enzyme treatment or thermocycling to exert controlled release of the USPIO cores. Light scattering and relaxation measurements were carried out on clustered and declustered MRS in aqueous solution. The work presented here offers a flexible platform for generating biocompatible, MR-visible nanomaterials with T2 relaxivities modulated by enzyme activity that presumably enable in vivo biosensing by modulation of image contrast.

## Results and Discussion

### Synthesis of PEG-PPS block copolymers and encapsulation of USPIO cores

**Figure 1:**
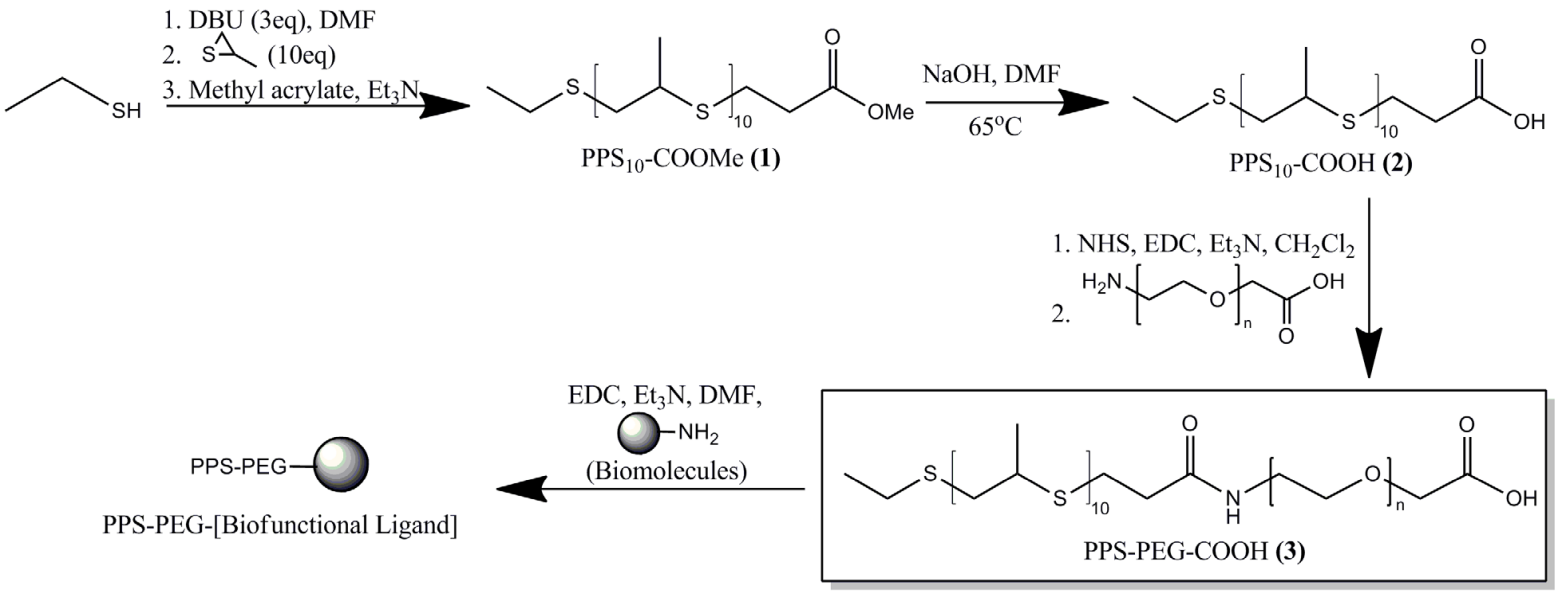
**Synthesis of PEG-PPS-based polymer-biomolecule conjugates**. The PPS block is formed by anionic ring-opening polymerization of an episulfide monomer, and endcapped with methyl acrylate. Conversion of the terminal methyl ester group to a carboxylic acid is accomplished under highly basic conditions to enable subsequent coupling of a PEG block and then a biofunctional ligand (e.g., peptides, amine-functionalized ssDNA) in modular fashion, yielding PEG-PPS-based polymer-biomolecule conjugates.

**Figure 2:**
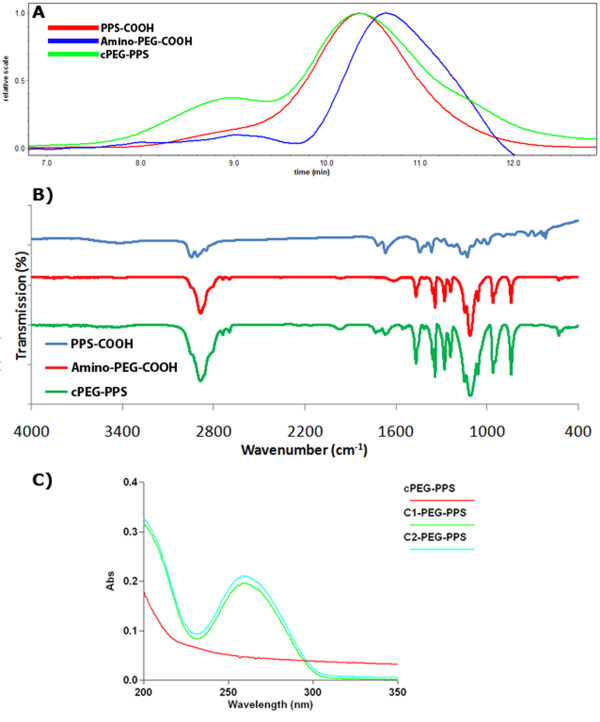
**Characterization of PEG-PPS copolymers**. (A) GPC-MALS characterization of PPS-COOH (red), H_2_N-PEG-COOH (blue), and cPEG-PPS (green). The cPEG-PPS sample displayed a population of polymers with peak elution time at ~9 min, corresponding to M_n _= 7.32 kDa (see Table 1), in addition to excess unreacted H_2_N-PEG-COOH. (B) FT-IR spectra of the same three polymer samples confirm the formation of the copolymer. The appearance of the 1700-1630 cm^-1 ^peak and disappearance of the 1650-1590 cm^-1 ^free amine bending peak in the copolymer versus the unreacted PEG is consistent with the formation of an amide linkage between the N-terminus of the PEG block and the C-terminus of the PPS block. (C) UV-Vis absorbance of cPEG-PPS (red) versus the post-dialyzed ssDNA-PEG-PPS conjugates (green, blue) confirms the conjugation of ssDNA to the polymers, as referenced by the characteristic peak at 260 nm.

The anionic ring opening polymerization scheme allows some degree of flexibility in fashioning PPS blocks with various functional groups on both ends of the polymer chain. This is done by varying the initiator and the chain terminator used in the reaction [34-38]. Ethanethiol was chosen as an initiator because the thiol is easily deprotonated by a small excess of DBU, without significant risk of the DBU leading to side products during the polymerization process (Figure 1). Injection of a stoichiometric amount of the propylene sulfide monomer forms the PPS block, and the reaction is endcapped with methyl acrylate via a Michael-type addition mechanism [35]. Hydrolysis of the terminal methyl ester under alkaline conditions fashioned PPS-COOH, at 1.65 kDa and PDI 1.18 (Table 1, Figure 2a). Acrylic acid was also investigated as an endcapping agent for the living polymerization in an attempt to fashion PPS-COOH in a single step, but resulted in undesirable side products that were likely formed by competing mechanisms to Michael-type addition (data not shown). Attachment of the PEG block and the ssDNA block were both done via well-characterized carbodiimide chemistry [19]. The construction of carboxylated PEG-PPS (cPEG-PPS) was confirmed via FT-IR (Figure 2b) and NMR spectroscopies, and further visualized by GPC, as the copolymers exhibited an elution peak centered at around 9 min that was not seen on the elution profiles of either of the precursor blocks (Figure 2a). Attachment of the ssDNA block was confirmed by UV-Vis spectrophotometry of extensively dialyzed PPS-PEG-ssDNA, characterized by the appearance of a peak at 260 nm for both ssDNA-coupled samples (Figure 2c).

**Table 1:** Molecular weight data for synthesized polymers.^a)^

Polymers	**dn/dc at 40°C **^**b)**^	***M***_***n***_	***M***_***w***_	PDI	***M***_***n ***_**from NMR**	Average Degree of polymerization by NMR
						
	mL/g	Da	Da	unitless	Da	
PPS-COOH	0.246	1650	1950	1.18	874	10
H_2_N-PEG-COOH ^c)^	--	4200	--	--	--	110
cPEG-PPS	0.183	7320	10200	1.39	5100	--

^a) ^Molecular weight of polymers was determined by GPC-MALS. Polymers were injected into a TSKGel Mixed Bed HZM-N column (4.6 mm ID × 15 cm) and chromatograms from the MALS detector and differential refractive index detector were used to analyze for polydispersity.

^b) ^dn/dc values were measured in offline batch mode by direct injection of serial dilutions of polymer samples into the refractive index detector of the GPC. The sample cell was maintained at 40°C. Data analysis was done on the Wyatt Astra software.

^c) ^Values for *M_n_*, *M_w_*, and PDI were provided by manufacturer.

With the PEG-PPS copolymers and the polymer-ssDNA conjugates complete, polymer-coated USPIO-core micelles were formed using the thin film hydration method [39]. In this process, a mixture of as-synthesized USPIO cores and polymers in toluene is completely dried by rotary evaporation, and then rehydrated to form micelles. In concept, the hydrophobic PPS blocks are expected to mingle with the oleic acid surfactant on the USPIO surface, with the PEG blocks and ssDNA extending into the surrounding aqueous medium, stabilizing the micelle. The micellization process resulted in a considerable amount of insoluble side products that can be easily precipitated away by magnet, leaving a colloidal phase that is then isolated into a fresh vial. Free, unloaded PEG-PPS was colloidally unstable and was easily removed by centrifugation, but the iron-containing micelles appeared stable in water and did not flocculate over several months. As little as 1.5:1 (w/w) ratio of polymer to iron oxide is sufficient to render PEG-PPS-USPIO micelles water-soluble. The micelles exhibited hydrodynamic diameters of 41 nm as measured by DLS. They appear so colloidally stable that they are extremely difficult to pellet without an ultracentrifuge, and are very slowly pelleted in proximity to a 1 T-field strength neodymium magnet. These observations have been suggested by other groups working with colloidal USPIOs [40-42].

**Figure 3:**
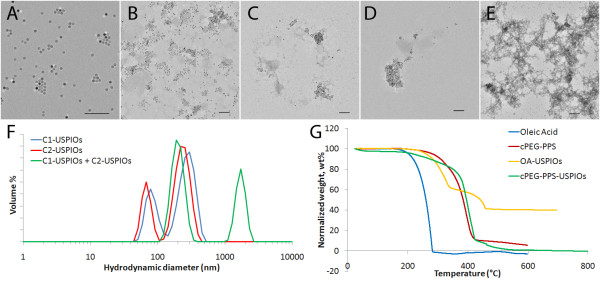
**Properties of functionalized USPIOs**. TEM of (A) as-made oleic acid-stabilized USPIO cores, (B) cPEG-PPS-USPIO micelles, (C) C1-USPIOs, (D) C2-USPIOs, and (E) clusters formed by hybridization of C1-USPIOs and C2-USPIOs. All scale bars are in 100 nm. In the first four cases, nanoparticles appear to be generally well-dispersed, but were also capable of forming short-ranged packing structures. (F) DLS size-volume distributions of C1-USPIOs (blue), C2-USPIOs (red), and clusters formed by hybridization of C1- and C2-USPIOs (green) suggest that the individual ssDNA-displaying USPIO micelles exhibit a hydrodynamic diameter of approximately 70 nm, while clusters formed by mixing the two populations are generally greater than of 1 μm in diameter. (G) TGA weight loss curves of oleic acid (blue), cPEG-PPS (red), OA-USPIOs (yellow), and cPEG-PPS-USPIOs (green) suggest that oleic acid is not displaced in the micellization process, and instead is encapsulated into the interior of the micelles along with the iron oxide core.

The morphology of the particles before and after encapsulation in PEG-PPS was assessed by TEM. As-made oleic acid-stabilized 12 ± 1 nm USPIO cores were deposited and dried on the TEM grid from toluene and generally appeared well-dispersed, but were also capable of forming short-ranged packing structures that showcased their monodispersity (Figure 3a). These same observations applied for the cPEG-PPS encapsulated USPIO micelles deposited and dried on the same copper TEM grids out of water (Figure 3b). The addition of PPS-PEG-ssDNA conjugates into the micellization process immobilized ssDNA on the USPIOs and also produced micelles of similar morphology (Figure 3c-d). These two populations of ssDNA-displaying USPIOs can be then mixed to form longer-range clusters that can be characterized by both TEM (Figure 3e) and DLS (Figure 3f). As shown in Figure 3f, free ssDNA-displaying USPIO micelles exhibited hydrodynamic diameters of ~70 nm (30 nm increase from the diameter exhibited by PEG-PPS-USPIO micelles is easily attributable to the length of the immobilized ssDNA sequences), while the clusters display diameters of upwards of 1 μm. The 100-200 nm peak picked up by the DLS is attributable to excess, unloaded non-iron-containing PEG-PPS micelles that remain present in the samples, even following several centrifugation steps as described above.

The presence of the PEG-PPS coating was investigated by thermogravimetric analysis (TGA) (Figure 3g). The precursor OA-USPIOs displayed evaporation profiles corresponding to oleic acid (250-350°C) and components of the iron oxide core (350-450°C). It is unclear why USPIO-associated oleic acid evaporated later than free oleic acid, although it is interesting to note that our results match those of other groups working on oleate-stabilized USPIOs [43]. cPEG-PPS-USPIO micelles also displayed two weight loss temperature ranges. The sharp evaporation range at 390-410°C can be attributed to the cPEG-PPS, while the long, gradual 200-390°C weight loss range is very likely made up of a combination of oleic acid evaporation, early cPEG-PPS desorption, and evaporation of iron oxide core components. This data suggests that the oleic acid surfactant remains anchored on the iron oxide cores during the micellization process with PEG-PPS, rather than being displaced in a ligand-exchange reaction. Taken together, this data suggests PEG-PPS-based copolymers and conjugates were capable of stably rendering water-soluble USPIOs displaying immobilized ligands to the surrounding environment.

### Clustering and de-clustering of complementary USPIOs leads to modulation of R_2 _relaxivity coefficients

R_2 _coefficients were calculated based on measurements of USPIO iron content through the phenanthroline assay [44] and relaxation time measurements. For all polymer-USPIO micelle samples, R_2 _values ranged between 400-500 mM^-1 ^s^-1^. These values were well within expected ranges, and are similar in order of magnitude to those recently measured by LaConte et al and Lee et al [45,46]. Differences in the absolute R_2 _values reported are easily accounted for, since LaConte et al used USPIO cores of smaller diameters (~6 nm), while Lee et al used USPIO cores that had been doped with other metals such as manganese.

When C1-USPIOs and C2-USPIOs were mixed, the hybridization of the surface-immobilized ssDNA sequences resulted in crosslinking of the USPIOs into larger clusters. This response is observed via an increase in hydrodynamic diameters from ~70 nm to above 1 μm (Figure 3f), and an increase in R_2 _coefficient to 690 ± 230 mM^-1 ^s^-1^. These effects of USPIO clustering on R_2 _are consistent with previously published results by Ai et al. [28]. Despite these previous demonstrations of this phenomenon, the mechanisms behind this "MRS" effect remain largely unstudied and are the subject of an ongoing study in our group.

Since clustering of the complementary C1-USPIOs and C2-USPIOs resulted in expected changes in R_2_, the next goal was to determine whether reversal of the clustering process would likewise correspondingly reverse the observed increase in R_2_. Irreversible and reversible 'declustering' of the USPIO complexes was achieved through enzymatic treatment and through thermocycling experiments, respectively.

**Figure 4:**
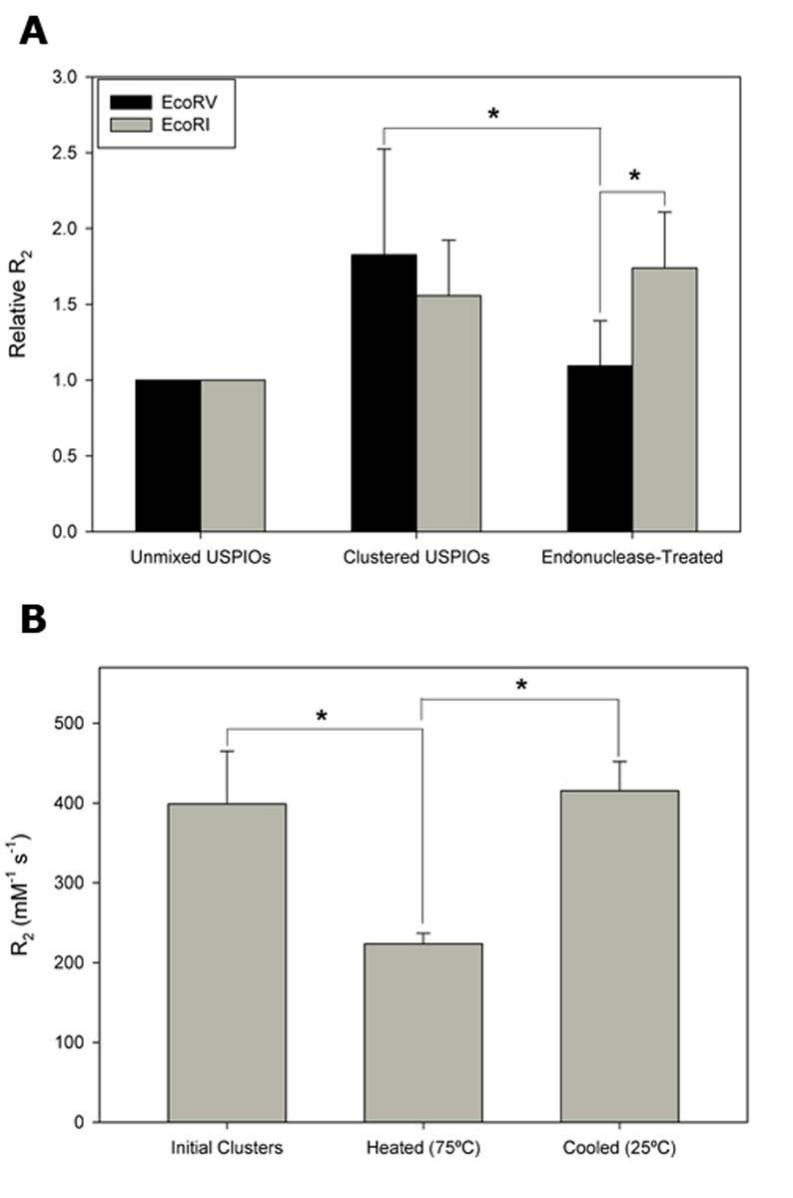
**Controlled release of USPIO micelles by environmental triggers**. (A) Self-assembly of EcoRV-sensitive ssDNA-USPIO clusters, and subsequent enzymatic treatment results in measurable changes in R_2 _relaxation coefficient relative to initial values. Following EcoRV treatment, R_2 _values return to baseline, a phenomenon that is in significant contrast to the effects of EcoRI treatment of the same clusters (n = 6). * p < 0.05. (B) Thermocycling of DNA-crosslinked USPIO clusters results in measurable changes in R_2 _relaxation coefficient. Heating of the clusters melts the DNA and results in declustering of the particles, corresponding to an approximately 50% decrease in R_2 _coefficient. After allowing the system to cool, the R_2 _coefficient increases to original levels, suggesting the reclustering of the ssDNA-USPIOs (n = 3). * p < 0.05.

By design, the hybridization of USPIO-immobilized C1- and C2- sequences reveals an EcoRV blunt end cleavage site. Treatment of the clusters with EcoRV is thus expected to redisperse the individual micelles, resulting in irreversible return of the R_2 _values to the baseline levels prior to the formation of the clusters. The rate of de-clustering is expected to be controlled by the relative concentrations of the enzyme and substrate. The 4-hour enzyme treatment in this proof-of-concept study allowed the de-clustering to go to completion. These expectations are confirmed by relaxometry data (Figure 4a), where a stable and significant difference in R_2 _(p < 0.05) is measured following treatment with EcoRV. The final R_2 _values remained stable for several hours following enzymatic treatment, suggesting that the declustering of the USPIOs was irreversible. In contrast, clusters were alternatively treated with EcoRI as a negative control, and the lack of a declustering response is reflected in an insignificant change in the R_2 _coefficient of the system.

Next, reversible declustering of the USPIOs was achieved through thermocycling, where R_2 _measurements were made while the USPIO clusters were being subjected to heating and cooling. In this process, heating and cooling of the clusters melts and reanneals the crosslinking DNA sequences, respectively. The resulting changes in the clustering of USPIOs leads to expected fluctuations in R_2 _(Figure 4b). The heated clusters are expected to decluster, resulting in the return of R_2 _values to baseline levels prior to mixing C1-USPIOs and C2-USPIOs. Allowing the system to cool is expected to reanneal the DNA sequences and reform clusters, returning R_2 _levels to the ranges expected for clusters. Our observations matched these expectations. Heating of the clusters resulted in approximately 50% decrease in R_2_, while return of the system to room temperature resulted in recovery of the original R_2_.

## Conclusions

Novel PEG-PPS based polymer conjugates were synthesized and characterized, then applied as a USPIO coating in the thin film hydration method to yield USPIO micelles. The synthesis of ssDNA-tagged polymers and the easy incorporation of these species into the micelle formation process leads to the facile formation of USPIO micelles that display biological ligands to the surrounding media. The generation of complementary populations of ssDNA-USPIOs results in a system that is capable of detecting enzymatic cleavage events through significant changes in R_2 _relaxation coefficient of the system. These results motivate ongoing studies in our group involving proteolytically-degradable USPIO clusters for the detection of matrix metalloproteinase activity in tumors.

## Methods

### General

All materials and reagents were purchased from Sigma-Aldrich (St. Louis, MO) and used as purchased unless otherwise specified. Methyl acrylate was purchased from Sigma-Aldrich (St. Louis, MO) and was purified by distillation prior to use. Heterobifunctional PEG reagents were purchased from Laysan Bio (Arab, AL) and used as purchased. The restriction enzymes EcoRI and EcoRV were purchased from New England Biolabs (Ipswich, MA). Custom ssDNA sequences designated C1 (5'-amino-ACGTACGTGATATCTGCATGCA-3') and C2 (5'-amino-TGCATGCAGATATCACGTACGT-3') were purchased from Sigma-Genosys. By design, C1 and C2 are complementary sequences that lack the ability to self-hybridize into hairpins and other undesired complexes.

Polymer samples were prepared for FT-IR spectroscopy by mixing with IR-grade KBr and pelleting on a KBr press. FT-IR was performed on a Bruker Tensor 27 system. ^1^H NMR spectra were obtained at 400 MHz using a 9.4 T Oxford magnet operated by a Bruker AV-400 console. The main NMR probe for the instrument is a 5 mm Z-gradient broadband inverse (BBI) probe with automatic tuning and matching capability (ATM). Gel permeation chromatography (GPC) was performed on a Tosoh Biosciences TSKGel SuperHZ-M mixed bed column (4 × 10^6 ^Da exclusion limit; DMF + 0.1 M LiBr mobile phase) incubated at 60°C with a Shimadzu SPD-10A UV detector and RID-10A refractive index detector (Shimadzu Scientific Instruments, Columbia, MD), and a Wyatt miniDAWN Treos multi-angle light scattering detector (MALS; Wyatt Technology, Santa Barbara, CA).

Transmission electron microscopy (TEM) was conducted on a Philips CM20 system. Carbon film-backed copper grids (Electron Microscopy Sciences, Hatfield, PA) were dipped into nanoparticle suspensions of interest and blotted dry. This process was repeated three times. Images were collected using a CCD camera with AMT Image Capture Engine software (Advanced Microscopy Techniques, Danvers, MA), and sizing of the particles was automated using a particle analyzer on ImageJ software. For nanoparticle micelles deposited from water, samples were dried in a vacuum desiccator for 2 h, and then counterstained with 3% uranyl acetate in water (Electron Microscopy Sciences, Hatfield, PA) for 30 s, gently blotted dry, and further dried in the vacuum desiccator for another 2 h prior to imaging.

For thermogravimetric analysis (TGA), samples were weighed as approximately 5 mg and deposited into a platinum pan for analysis with the Instrument Specialist's TGA-1000 (Instrument Specialists, Inc., Twin Lakes, WI). Samples were heated to approximately 200 K past their expected vaporization point and were heated at a rate of 10 K per minute.

### Synthesis and characterization of oleic acid-coated USPIO cores

Synthesis of USPIO cores was done based on the procedures described by Woo et al. [14]. Under argon gas flow, oleic acid (3.8 mL, 12 mmol) was heated to 100°C in 40 mL octyl ether in a three-neck flask. Fe(CO)_5 _(0.8 mL, 6 mmol) was then injected into the system, and the mixture was then refluxed at 280°C for 4 h. Next, the mixture was cooled to 80°C and aerated overnight (> 14 h). The mixture was then refluxed for 2 h at 280°C and then cooled back to room temperature. Oleic acid-stabilized USPIOs (OA-USPIOs) were collected following three washes in ethanol and centrifugation, and air dried overnight to form a dark brown-black powder.

### Synthesis of PPS-COOMe (1)

The PPS block was synthesized via anionic ring opening polymerization of propylene sulfide from a deprotected ethanethiol initiator (Figure 1). To form the initiator, 3 eq of the deprotectant 1,8-diazabicycloundec-7-ene (DBU; 11.2 mL; 75 mmol) was mixed in 40 mL dry DMF in a Schlenk tube, followed by the addition of 1 eq of ethanethiol (1.85 mL; 25 mmol). The tube was evacuated via a membrane pump and equilibrated with argon 6×, and then stirred at room temperature for 10 min. Monomer was then added to the vessel by injection of 10 eq propylene sulfide (19.6 mL; 250 mmol) into the vial, and polymerization occurred for 90 min. In a separate Schlenk tube, 10 eq distilled methyl acrylate (22.5 mL; 250 mmol) was mixed with 5 eq triethylamine (Et_3_N; 17.4 mL; 125 mmol). This vial was evacuated via a membrane pump and equilibrated with argon gas 6×, and then the contents were transferred under vacuum into the PPS-containing vial. Upon mixing of the two liquids, a color change is observed from orange to yellowish. This mixture was then left to stir overnight at room temperature. Concentrated product was obtained by removal of DMF under high vacuum, and was redissolved in CH_2_Cl_2 _(100 mL). This solution was extracted 7 times in brine. The collected organic phase was then dried over 5 g of sodium sulfate, and residual salts were removed by gravity filtration through a #5 Whatman filter disc. The product was concentrated by incomplete evaporation of the CH_2_Cl_2 _under vacuum, and then precipitated by addition to ice-cold hexanes for 30 min. Centrifugation for 5 min at 800 × g pellets the PPS block, and the hexane extraction step and centrifugation was repeated a second time to yield PPS_10_-COOMe (PPS-COOMe). Average degree of polymerization was estimated by NMR. FT-IR (KBr) 1737 (s, ester C = O), 1490-1400 (t, C-H from PPS block and ethyl terminus overlapped), 693 (s, CH_2_-S). *δ*_H _(400 MHz; CDCl_3_): *δ *1.2-1.3 (t, C**H_2 _**next to carboxylic terminus), 1.3-1.4 (d, C**H_3 _**in PPS block & terminal C**H_3_**), 2.5-2.8 (broad s, C**H **in PPS block), 2.8-3.1 (broad s, C**H_2 _**next to S), 3.72 (s, C**H_3 _**in ester).

### Synthesis of PPS-COOH (2)

PPS_10_-COOH was synthesized from PPS_10_-COOMe (1) by mixing the latter in 0.1 M NaOH in DMF at 65°C for 5 h under open air in a fume hood (Figure 1). This setup drives the reaction forward as the MeOH byproduct evaporates directly into the environment. After the reaction was cooled to room temperature, concentrated product was obtained by evaporation of DMF under high vacuum, and was redissolved in CH_2_Cl_2 _(100 mL). This solution was extracted 7 times in brine. The collected organic phase was then dried over 5 g of sodium sulfate, and residual salts were removed by gravity filtration through a #5 Whatman filter disc. The product was concentrated by incomplete evaporation of the CH_2_Cl_2 _under vacuum, and then precipitated by addition to ice-cold hexanes for 30 min. Centrifugation for 5 min at 800 × g pellets the PPS block, and the hexane extraction step and centrifugation was repeated a second time to yield PPS_10_-COOH (PPS-COOH), a viscous yellow liquid, at more than 90% conversion, as confirmed by NMR spectroscopy. The carboxylic acid terminus remains unprotonated, as confirmed by the lack of the corresponding proton peak on FT-IR and NMR. FT-IR (KBr) 1737 (s, ester C = O; incomplete ester hydrolysis), 1713 (s, carboxylic C = O), 1490-1400 (t, C-H_3 _and C-H_2 _overlapped), 693 (s, CH_2_-S). *δ_H _*(400 MHz; CDCl_3_): *δ *1.2-1.3 (t, C**H_2 _**next to carboxylic terminus), 1.3-1.4 (d, C**H_3 _**in PPS block & terminal C**H_3_**), 2.5-2.8 (broad s, C**H **in PPS block), 2.8-3.1 (broad s, C**H_2 _**next to S).

### Synthesis of cPEG-PPS (carboxylated PEG-PPS; 3) and PPS-PEG-ssDNA conjugates

PPS_10_-COOH (2), 2 g was dissolved into 3 mL of CH_2_Cl_2 _and reacted with ~5 eq of *N*-hydroxysuccinimide (NHS; 1.44 g; 12.5 mmol), 1-Ethyl-3-(3-dimethylaminopropyl) carbodiimide hydrochloride (EDC; 2.40 g; 12.5 mmol), and Et_3_N (1.74 mL; 12.5 mmol) with gentle vortexing for 4 h at room temperature. Following the reaction, the crude product was concentrated by rotary evaporation. Excess salts were precipitated and extracted 2× with brine and 3× with deionized water, and the product was dried by rotary evaporation. 1 mL of the product was redissolved in 5 mL of DMF, and then reacted with ~0.1 eq of *M_n _*5 kDa H_2_N-PEG-COOH (625 mg; ~125 μmol) in the presence of 0.2 eq of Et_3_N (34 μL; 250 μmol) overnight. The crude product was concentrated by rotary evaporation, dissolved in 10 mL CH_2_Cl_2_, and precipitated twice in diethyl ether under ice for 1 h in order to remove unreacted PPS. Excess organic solvents were removed by rotary evaporation, and the crude product was dissolved in deionized water and rinsed in 100 kDa MWCO centrifugal filters (Corning Life Sciences, Lowell, MA) with six fill volumes of deionized water, to remove unbound PEG. Lyophilization of the product overnight yielded cPEG-PPS (carboxylated PEG-PPS). *δ*_H _(400 MHz; CDCl_3_): *δ *1.2-1.3 (t, C**H_2 _**next to carboxylic terminus), 1.3-1.4 (d, C**H_3 _**in PPS block & terminal C**H_3_**), 1.4-1.8 (broad s, C**H_2 _**next to COOH), 2.5-2.8 (broad s, C**H **in PPS block), 2.8-3.1 (broad s, CH_2 _next to S), 3.6-3.8 (s, C**H_2 _**in PEG block), 4.18 (s, N**H **in amide bond).

To construct the ssDNA-PEG-PPS conjugates, the 5'-aminated custom ssDNA sequences C1 and C2 were each separately reacted with cPEG-PPS in 5 mL sequencing grade DMF in scintillation vials. 1.5 μmol of each ssDNA sequence was transferred to each DMF-containing vial in 0.5 mL NaCl buffer in water. Following addition of equimolar amounts of cPEG-PPS, 5 eq of EDC and Et_3_N were added to the reactions. The mixtures were briefly bubbled with argon gas, then capped and vortexed for 2 h at room temperature. Following rotary evaporation to remove excess DMF and Et_3_N, the crude products were dissolved in DNAse-free water (Sigma-Aldrich) and dialyzed separately in 30 kDa MWCO centrifugal filters (Corning Life Sciences, Lowell, MA) with ten fill volumes of DNAse-free water. The presence of DNA-polymer conjugates was confirmed by the appearance of a 260 nm absorbance peak versus unreacted cPEG-PPS, by UV-Vis spectrophotometry.

### Encapsulation of USPIO core nanoparticles with polymers

USPIO-core, polymer-shell micelles/nanoparticles were formed by the thin-film hydration method [39]. Briefly, 15 mg of purified PEG-PPS-based polymers were dissolved with 10 mg of OA-USPIOs in 1 mL toluene, vortexed to mix, sonicated for 5 s to break apart clumps, and then dried by rotary evaporation for 20 min. The dried polymer/USPIO mixture was then rehydrated in 5 mL of DNAse-free water and vortexed vigorously to suspend all particulates. Large clumps and byproducts are easily removed by magnetic pelleting, and the colloidal phase is collected and further centrifuged at 2500 × g for 5 min to precipitate excess polymers. The supernatant is gently aspirated by pipet into fresh scintillation vials and stored at 4°C.

### Phenanthroline assay for iron content determination

To quantify the concentration of iron in all PEG-PPS-USPIO formulations, the 1,10-phenanthroline assay was used [44]. USPIOs in PBS (50 μL) were mineralized by treatment in concentrated H_2_SO_4 _for 30 min at room temperature, resulting in a loss of the dark brownish-black color of the solution. This was followed by treatment of the mixture with 10 μL 100 mg/mL hydroxylammonium chloride in water and 50 μL 1 mg/mL 1,10-phenanthroline in water. Development of an intense orange color, corresponding to the presence of iron, is observed upon addition of 550 μL 100 mg/mL sodium acetate in water. Absorbance at 510 nm was measured on a Varian Cary 50 UV-Vis-NIR spectrophotometer (Palo Alto, CA). The concentration of free iron was calculated based on a standard curve constructed using serial dilutions of ferrous ammonium sulfate (Fisher Scientific, Pittsburgh, PA) in water. Measurements of each sample were done in triplicate.

### R2 relaxation measurements

A 0.5 T Maran tabletop NMR scanner with DRX-II console (Oxford Instruments, Oxfordshire, UK) was used for transverse (T_2_) relaxation time measurements. 200 μL of PEG-PPS-USPIOs in PBS were loaded into 5-mm thin-walled NMR tubes and introduced into the scanner. Measurements were made using a Carr-Purcell-Meiboom-Gill (CPMG) sequence at room temperature, 32 echoes with 12 ms time between echoes, and an average of 9 acquisitions. R_2 _relaxation coefficients were calculated based on the following formula [46], where [Fe] is the iron content of the sample as determined through the phenanthroline assay (described earlier):

(1) $$R_{2}=\frac{1}{T_{2}\times [Fe]}$$

For clustering/declustering experiments, 100 μL of complementary ssDNA-USPIO populations were mixed in the NMR tubes and allowed 10 min to cluster before T_2 _was remeasured as described above. To study the effects of restriction enzyme treatment, 500 U of EcoRI or EcoRV were added to the tubes according to the manufacturer's instructions and the system was incubated at 37°C for 4 h before relaxation time was remeasured. To study the effects of thermocycling, samples in NMR tubes were heated to 85°C in a water bath for 15 min, then measured in the relaxometer. The temperatures in heated ssDNA-USPIO samples did not drop below 70°C during the measurement process. Unless otherwise noted, all presented data is the average of three independent experiments. Statistical significance was established using the paired Student's t-test for all samples.

## Competing interests

The authors declare that they have no competing interests.

## Authors' contributions

SSY and RLS planned and carried out all polymer synthesis, characterization, and micelle synthesis and characterization, with extensive input from TDG. RAO carried out all gravimetric work and repeated relaxation measurements. CSB performed all NMR work and aided in analysis. CPO and JAH contributed extensive technical consultation and expertise on the properties and synthesis of the block copolymers used in this study. All authors have read and approved the final manuscript.
