# Enzymatic- and temperature-sensitive controlled release of ultrasmall superparamagnetic iron oxides (USPIOs)

**DOI:** 10.1186/1477-3155-9-51

**Published:** 2011-11-14

**Authors:** Shann S Yu, Randy L Scherer, Ryan A Ortega, Charleson S Bell, Conlin P O'Neil, Jeffrey A Hubbell, Todd D Giorgio

**Affiliations:** 1 Department of Biomedical Engineering, Vanderbilt University; Nashville, Tennessee, USA; 2 Vanderbilt Institute for Nanoscale Science and Engineering, Vanderbilt University; Nashville, Tennessee, USA; 3 Interdisciplinary Program in Materials Science, Vanderbilt University; Nashville, Tennessee, USA; 4 Integrative Biosciences Institute, École Polytechnique Fédérale de Lausanne, Lausanne, Switzerland

## Correction

After publication of this work [1], we found an error in the figure legend for Figure two. In Figure 2A, GPC chromatograms of the synthesized co-polymers were shown, but the completed cPEG-PPS product contains some residual PPS-COOH, not H_2_N-PEG-COOH as originally described in the figure legend.
